# Technology-Based Alcohol Interventions in Primary Care: Systematic Review

**DOI:** 10.2196/10859

**Published:** 2019-04-08

**Authors:** Alex T Ramsey, Jason M Satterfield, Donald R Gerke, Enola K Proctor

**Affiliations:** 1 Department of Psychiatry Washington University School of Medicine St Louis, MO United States; 2 Department of Medicine University of California San Francisco San Francisco, CA United States; 3 Graduate School of Social Work University of Denver Denver, CO United States; 4 Brown School of Social Work Washington University in St Louis St Louis, MO United States

**Keywords:** alcohol drinking, risky health behavior, alcohol-related disorders, internet, computers, mobile health, primary health care, implementation science, review

## Abstract

**Background:**

Primary care settings are uniquely positioned to reach individuals at risk of alcohol use disorder through technology-delivered behavioral health interventions. Despite emerging effectiveness data, few efforts have been made to summarize the collective findings from these delivery approaches.

**Objective:**

The aim of this study was to review recent literature on the use of technology to deliver, enhance, or support the implementation of alcohol-related interventions in primary care. We focused on addressing questions related to (1) categorization or target of the intervention, (2) descriptive characteristics and context of delivery, (3) reported efficacy, and (4) factors influencing efficacy.

**Methods:**

We conducted a comprehensive search and systematic review of completed studies at the intersection of primary care, technology, and alcohol-related problems published from January 2000 to December 2018 within EBSCO databases, ProQuest Dissertations, and Cochrane Reviews. Of 2307 initial records, 42 were included and coded independently by 2 investigators.

**Results:**

Compared with the years of 2000 to 2009, published studies on technology-based alcohol interventions in primary care nearly tripled during the years of 2010 to 2018. Of the 42 included studies, 28 (64%) were randomized controlled trials. Furthermore, studies were rated on risk of bias and found to be predominantly low risk (n=18), followed by moderate risk (n=16), and high risk (n=8). Of the 24 studies with primary or secondary efficacy outcomes related to drinking and drinking-related harms, 17 (71%) reported reduced drinking or harm in all primary and secondary efficacy outcomes. Furthermore, of the 31 studies with direct comparisons with treatment as usual (TAU), 13 (42%) reported that at least half of the primary and secondary efficacy outcomes of the technology-based interventions were superior to TAU. High efficacy was associated with provider involvement and the reported use of an implementation strategy to deliver the technology-based intervention.

**Conclusions:**

Our systematic review has highlighted a pattern of growth in the number of studies evaluating technology-based alcohol interventions in primary care. Although these interventions appear to be largely beneficial in primary care, outcomes may be enhanced by provider involvement and implementation strategy use. This review enables better understanding of the typologies and efficacy of these interventions and informs recommendations for those developing and implementing technology-based alcohol interventions in primary care settings.

## Introduction

### Background

Alcohol use is a leading risk factor for global disease burden, and recent findings indicate that even light-to-moderate drinking is detrimental to all-cause mortality [1,2]. Additionally, approximately 14% of US adults annually, and nearly 30% for lifetime, engage in harmful drinking consistent with alcohol use disorder [3]. This disorder contributes to over 200 diseases and health problems, including cirrhosis, cancers, fetal alcohol syndrome, assaults, and crash-related fatalities, with costs totaling US $249 billion annually in the United States alone [4-6]. Primary care settings that integrate physical and behavioral health care are uniquely situated to reach this at-risk population through delivery of evidence-based interventions (EBIs) to reduce harmful alcohol use [7,8].

To enhance the capacity for delivering behavioral health services, new approaches—such as those provided by digital technologies—can assess and intervene to reduce alcohol use (and associated harm) and facilitate referrals to specialty treatment [9]. In fact, health centers are increasingly leveraging technology to reduce medical staff burden, facilitate electronic health record (EHR) integration, improve standardization and fidelity, and enhance service efficiency [10,11]. Additionally, recent randomized controlled trials have begun to highlight the promise of using technology-supported platforms—including computers, kiosks, or tablets—to deliver efficacious alcohol-related interventions in primary care [7,11,12]. Relatedly, telephone-delivered interventions, although less novel, remain popular approaches in primary care, despite a lack of synthesized research on effectiveness and optimal implementation.

Systematic reviews exist for generalized alcohol interventions in primary care [13,14] and technology-based behavioral health interventions in other settings [15-17]. However, despite emerging effectiveness data in both adult and adolescent populations [17], few efforts have been made to summarize the collective findings of technology-based alcohol interventions in primary care settings. In total, 2 excellent Cochrane reviews on the effectiveness of brief alcohol interventions were recently published; one was based in primary care but did not focus on technology-based interventions [18] and the other focused on technology-based interventions but the focus was largely outside of primary care settings [19]. Another systematic review examined digital and computer-based alcohol interventions in primary care [20]; however, this review includes a broader set of technology-based interventions, addresses important effect modifiers, and provides a substantial update, adding 4 years (2015 to 2018) beyond the previous review. Use of technology in primary care is rapidly evolving, and systematically updating the collective knowledge gained from recent efforts in this area is needed to inform future delivery of technology-based alcohol interventions in these settings.

Furthermore, although technology-based behavioral health interventions are likely to be diverse in nature, there is currently a lack of conceptual clarity and no system for categorizing these interventions (eg, patient-facing, provider-facilitated, or a combination of both), making it difficult to directly compare interventions with similar purposes and approaches. The conceptualization and categorization provided here is useful for intervention developers and researchers in this area of investigation. The overarching purpose of this study was to review the most recent literature on the use of technology to deliver, enhance, or support the implementation of alcohol-related interventions in primary care.

### Research Questions

In this review, we focused on addressing the following key questions regarding technology-based alcohol interventions in primary care:

1. What proportion of technology-based alcohol interventions is delivered (1) directly to the patient, (2) by the provider via technology-based medium, (3) some combination of both, or (4) directly to the provider to improve care?
2. What factors supported the use of technology-based alcohol interventions (technological platforms, delivery contexts, implementation strategies, and EHR integration)?
3. What proportion of studies reported that the technology-based intervention (1) reduced drinking or alcohol-related harms and (2) demonstrated superiority to treatment as usual (TAU)?
4. Did efficacy differ by the (1) context in which the intervention was delivered, (2) type of technology, (3) categorization or target of the technology-based intervention, and (4) use of an implementation strategy?

Answers to these questions would provide researchers with a better system of categorizing a diverse set of technology-based interventions and an understanding of the efficacy and effect modifiers of these interventions. These important contributions would inform recommendations for those developing and implementing technology-based alcohol interventions in primary care settings.

## Methods

### Search Strategy

In line with the Preferred Reporting Items for Systematic reviews and Meta-Analyses Statement and supporting publications to enhance the rigor of systematic reviews [21,22], we conducted a systematic literature review of English-language publications on completed research studies from January 2000 to December 2018. In total, 4 EBSCO databases (CINAHL Plus, Global Health, MEDLINE, and PsycInfo), ProQuest Dissertations, and Cochrane Reviews were searched. Although a comprehensive search of gray literature was not feasible, we did include dissertations and search trial registries (eg, PROSPERO). A total of 77 Boolean search terms were used to identify articles at the intersection of primary care, technology, and alcohol-related problems (see Multimedia Appendix 1). In conjunction with 2 university research librarians, extensive testing was conducted to limit the number of articles outside the inclusion criteria and yet ensure that the search strategy yielded comprehensive results. The systematic review protocol was published and is accessible on PROSPERO, an international prospective register of systematic reviews.

**Figure 1:**
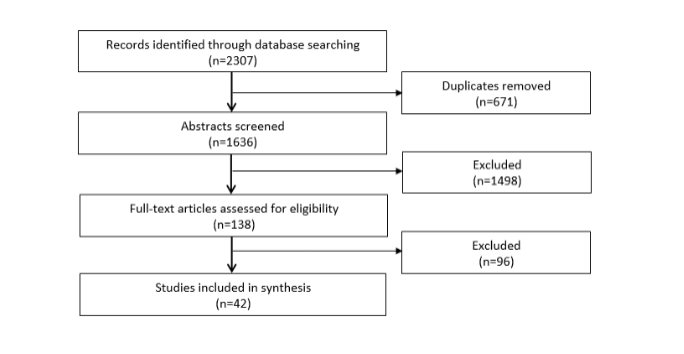
Flow diagram of search and screening results.

### Study Selection

As illustrated in Figure 1, the initial database search yielded 2307 records, which reduced to 1636 records after removing duplicates. One of the 2 study investigators trained in conducting systematic reviews (AR or DG) reviewed the title and abstract of each record to assess study eligibility, and full text was obtained when appropriate. Studies were excluded if the article title or abstract did not specify that the study included a technology-based intervention that focused on alcohol use and was delivered in a primary care setting. A total of 138 studies were identified for full-text review and final selection for data abstraction.

Both study investigators (AR and DG) then independently reviewed each full text article for final inclusion, achieving an interrater reliability of .84. All initial discrepancies were resolved mutually through discussion between the same 2 investigators. Studies were excluded during this step if they were a self-defined pilot study or only reported on feasibility outcomes, alcohol use outcomes were not reported separately from other health outcomes, the so-called intervention only constituted screening for alcohol misuse, or the study results had previously been reported elsewhere (eg, main study outcome article). On the basis of this full-text review, 42 studies met our final inclusion criteria for coding [12,23-63].

### Data Abstraction and Analysis

All data from included articles were recorded using a standardized data abstraction form, which both study investigators (AR and DG) completed independently, again mutually resolving all initial discrepancies through discussion. To address our key questions, articles were coded on a range of topics, including study design and sample size, type of technology, category or target of intervention, location of delivery, implementation strategies used, primary and secondary outcomes, intervention efficacy results, and a risk of bias score to inform study quality (see Multimedia Appendix 2). The wide heterogeneity of primary outcomes and assessment tools precluded the ability to conduct a meta-analysis; instead, data from the articles were primarily summarized descriptively. However, we conducted one-way analyses of variance (ANOVAs) with an efficacy score treated as the dependent variable and several of the factors listed above—location of delivery (ie, delivery context), type of technological platform used, category of intervention, and presence of an implementation strategy—treated as the independent variables.

## Results

### Descriptive Analyses

Of the 42 included studies, 5 (12%) studies were published from 2000 to 2004, 7 (17%) were published from 2005 to 2009, 21 were (50%) published from 2010 to 2014, and 9 (21%) were published from 2015 to 2018. The included studies were conducted in 8 different countries, with 33 of 42 studies (79%) based in the United States. Of the 42 included studies, 28 (64%) featured randomized designs, 10 (24%) were quasi-experimental, and 4 (10%) were observational studies. Multimedia Appendix 2 presents further information on designs for each study.

### Type of Technology

Technology-based interventions were also categorized by the technological platform used to deliver the intervention. Of the 47 technological platforms identified, 13 (28%) were telephone or telehealth, 13 (28%) were stand-alone computer or software, 10 (21%) were Web-based, 5 (11%) were mobile (eg, tablet and smartphone), 3 (6%) were interactive voice response, 2 (4%) were kiosk, and 1 (2%) was video.

### Category or Target of Intervention

Using a typology informed by research on behavior change at multiple levels [64], technology-based interventions were categorized into 4 main types: (1) *Patient-facing*; (2) *Provider-facilitated*; (3) *Patient-facing plus provider-facilitated*; and (4) *Provider-directed*. Table 1 summarizes these types of technology-based interventions, including the conceptual definitions and representative examples of each type. The coding team determined that, of the 42 included studies, 14 (33%) interventions were patient-facing, 11 (26%) were provider-facilitated, 11 (26%) were patient-facing plus provider-facilitated, and 6 (14%) were provider-directed.

### Delivery Context

Articles were coded into 3 broad delivery contexts: in clinic (eg, waiting room and exam room), out of clinic (eg, home and work), or both in and out of clinic (eg, part in waiting room and part at home). Of the 42 included articles, 18 (43%) reported interventions delivered in clinic, 17 (40%) reported interventions delivered at home (or otherwise outside of the clinic setting), and 7 (17%) reported interventions delivered both in clinic and at home.

### Implementation Strategies

Implementation strategies, conceptualized as “methods or techniques used to enhance the adoption, implementation, and sustainability of a clinical program or practice” [65], constitute an important component to delivery of evidence-based practices [66,67], including those facilitated by the use of technology [68,69]. Previous research has categorized implementation strategies into the following categories: planning, educating, financing, restructuring, managing quality, and attending to the policy context [70]. Although underreported and underspecified in clinical research [65], implementation strategies are necessary to maximize the translation of research-based interventions into practice settings [71,72]. For this review, we only considered approaches to be implementation strategies when health care professionals (rather than research staff) were involved in efforts to improve uptake or delivery of the intervention. Of the 42 studies included, 15 (36%) specified the use of an implementation strategy to support the delivery of technology-based alcohol interventions (see Multimedia Appendix 2).

We documented 17 total and 11 unique implementation strategies using the Expert Recommendations for Implementing Change compilation [71]. The reported implementation strategies included the following: *Conduct ongoing training* (n=3), *Make training dynamic* (n=3), *Provide ongoing consultation* (n=2), *Remind clinicians* (n=2), *Relay clinical data to providers* (n=2), *Facilitation* (n=2), *Develop educational materials* (n=1), *Organize clinician implementation team meetings* (n=1), *Prepare* *patients/consumers to be active participants* (n=1), *Develop a formal implementation blueprint* (n=1), *Assess for readiness* (n=1), and *Conduct cyclical small tests of change* (n=1). These strategies varied widely in type and intensity, ranging from in-depth training on use of the technology-based intervention to the use of reminder cards and posters for providers. Implementation strategies also included providing frequent supervision, comparison between health care staff versus self (patient)-referral to the technology-based intervention and linking clinical management to the technology-based platform. Of note, only 6 of 42 articles (14%) mentioned any type of integration between the technology-based intervention and existing EHR systems.

### Risk of Bias

We used a common classification scheme [73] to rate risk of bias pertaining to selection (eg, allocation concealment), performance (eg, blinding), detection (eg, validity of outcome assessment), attrition (eg, withdrawal rates), and reporting (eg, selective outcome reporting). We judged each of the 5 types of bias to be high (2), unclear (1), or low (0), and then calculated a sum risk of bias score on a scale of 0 to 10. Studies were then determined to have low (0 to 1), moderate (2 to 4), or high (5 to 10) overall risk of bias. Of the 42 studies, 18 (43%) were rated to be low risk of bias, 16 (38%) moderate risk of bias, and 8 (19%) high risk of bias.

### Primary and Secondary Outcomes

We classified primary and secondary efficacy outcomes into 7 categories: quantity of alcohol use (eg, number of drinks per drinking day), frequency of alcohol use (eg, total number of drinking days), severity of alcohol use or risk scores (eg, Alcohol Use Disorders Identification Test or Alcohol, Smoking and Substance Involvement Screening Test score), binge or heavy episodic drinking (eg, number of binge episodes in the past week), status of at-risk alcohol use (eg, proportion of individuals with categorically defined at-risk drinking), any use (proportion of individuals with any past 90-day alcohol use), and drinking consequences (academic or legal problems related to drinking). Multimedia Appendix 3 organizes results across these categories of outcomes for each of the 4 technology types.

**Table 1:** Types of technology-based alcohol interventions.

Type	Conceptualization	Examples
Patient-facing	Intervention is delivered directly to the patient via technology with very limited or no provider involvement	Stand-alone touchscreen kiosk-based brief intervention
Provider-facilitated	Intervention is delivered by provider to patient via technology-based medium	Telephone-based brief intervention
Patient-facing plus provider-facilitated	Packaged intervention that has at least one patient-facing component and at least one provider-facilitated component	Tablet-based screening and brief intervention plus telephone-based counseling
Provider-directed	Intervention is delivered to provider to improve or support patient care delivery	Web-based training and clinical management dashboard

### Efficacy of Intervention

Multimedia Appendix 3 summarizes study results according to statistical significance on 2 key outcomes—whether the intervention reduced drinking or drinking-related harms and whether the benefits were superior in comparison with TAU—for each primary and secondary outcome of each study. Of note, several studies reported multiple primary and secondary outcomes. At the study level, we assessed the proportion of primary and secondary efficacy outcomes that indicated reduced harm and that were determined to be superior to TAU. Similar methods of operationalizing and summarizing intervention efficacy have been used in previous systematic reviews in lieu of meta-analytic procedures [74].

Of the 24 studies with primary or secondary efficacy outcomes related to drinking and drinking-related harms, all 24 (100%) indicated reduced drinking or harm in at least half of the primary and secondary outcomes and 17 (71%) indicated reduced drinking or harm in all of the primary and secondary outcomes. Of the 31 studies with direct comparisons with TAU, 16 (52%) indicated that none of the primary and secondary outcomes were superior to TAU. However, of these 31 studies, 13 (42%) indicated that at least half of the primary and secondary outcomes were superior to TAU, and 8 (26%) indicated that all the primary and secondary outcomes were superior to TAU.

In examining predictors of intervention efficacy, we used the outcome of whether at least half of the primary and secondary efficacy outcomes were determined to be superior to TAU to maximize variability in the outcome, use a sufficiently rigorous cutoff, and focus on studies with direct comparisons with TAU. Of the 31 studies comparing intervention to TAU, 6 of 16 (38%) low risk of bias studies, 5 of 13 (38%) moderate risk of bias studies, and 2 of 2 high risk of bias studies reported at least half of their primary and secondary efficacy outcomes to be superior to TAU. This outcome was uncorrelated with the risk of bias (*P*=.812) variable.

### Predictors of Efficacy

Descriptive analyses and one-way ANOVAs were used to examine intervention efficacy based on (1) whether the intervention was delivered in the clinic, at home, or both in the clinic and at home, (2) the type of technology used (computer or Web, telephone or video, and mobile), (3) the category or target of intervention (eg, patient-facing), and (4) the specification of an implementation strategy.

1. *Delivery context.* Of the 31 studies comparing intervention to TAU, 3 of 14 (21%) studies in the clinic, 5 of 10 (50%) studies at home, and 5 of 7 (71%) studies both in the clinic and at home showed at least half of the outcomes superior to TAU. The level of intervention efficacy did not differ significantly based on whether the intervention was conducted in the clinic (mean 0.21 (SD 0.43)), at home (mean 0.50 (SD 0.53)), or both in the clinic and at home (mean 0.71 (SD 0.49); *F*_2,28_=2.81; *P*=.077). However, it should be noted here that there were only 7 cases in the category of both in the clinic and at home.
2. *Type of technology*. Of the 34 technological platforms within the 31 studies comparing intervention to TAU, 6 of 17 (35%) studies of computer or Web-based interventions, 7 of 13 (54%) telephone or video-based interventions, and 2 of 4 (50%) mobile-based interventions showed at least half of the outcomes superior to TAU. The level of intervention efficacy did not differ significantly based on whether the technology-based alcohol intervention was delivered via computer or Web (mean 0.35 (SD 0.49)), telephone or video (mean 0.54 (SD 0.52)), or mobile (mean 0.50 (SD 0.58); *F*_2,31_=0.51; *P*=.603).
3. *Category of intervention* *.* The first analysis examined the level of intervention efficacy for each separate category of intervention; the second analysis examined the level of intervention efficacy between interventions that were only patient-facing versus those that had a provider-based component. Of the 31 studies comparing intervention with TAU, 2 of 12 (17%) patient-facing, 6 of 9 (67%) provider-facilitated, 5 of 9 (56%) patient-facing plus provider-facilitated, and 0 of 1 provider-directed studies showed at least half of their intervention outcomes superior to TAU. There were no significant differences in intervention efficacy between the individual categories of patient-facing (mean 0.17 (SD 0.39)), provider-facilitated (mean 0.67 (SD 0.50)), patient-facing plus provider-facilitated (mean 0.56 (SD 0.53)), and provider-directed, yet the results trended toward significance such that the patient-facing interventions showed lower efficacy than the other groups; (*F*_3,27_*=* 2.54; *P*=.078). Indeed, when collapsing the groups that included a provider component and comparing them with patient-only interventions, we found that intervention efficacy was significantly higher for interventions that had a provider-based component (mean 0.58 (SD 0.51)) than for those that were patient-facing only (mean 0.17 (SD 0.39); *F*_1,29_=5.76; *P*=.023, adjusted R^2^=0.14 [medium effect size]).
4. *Specification of implementation strategy* *.* As implementation strategies were conceptualized as methods to improve the delivery of a clinical intervention, the provider-directed technologies were considered synonymous and indistinguishable from implementation strategies. Implementation strategies were analyzed as effect modifiers for the other types of interventions (eg, patient-facing); therefore, it was determined inappropriate to include the provider-directed technologies in this analysis as the intervention would have been the same as the effect modifier. Therefore, of the 31 studies comparing intervention with TAU, the provider-directed study (n=1) was removed for this particular analysis. In the remaining 30 studies, 6 of 6 (100%) studies reporting use of an implementation strategy showed at least half of the outcomes superior to TAU versus 7 of 24 (29%) studies not reporting use of an implementation strategy. Intervention efficacy was significantly higher when an implementation strategy was employed to facilitate delivery of the intervention (mean 1.00 (SD 0.00)) than for those with no specified implementation strategy (mean 0.29 (SD 0.46); *F*_1,28_=13.60; *P*=.001; adjusted R^2^=0.30 [large effect size]).

## Discussion

### Principal Findings

The use of technology-facilitated interventions in primary care settings is a burgeoning issue in behavioral health; however, research-based guidance is needed to inform development and implementation to ensure that these tools enhance, rather than impede, the efficiency and effectiveness of alcohol interventions in this setting. Our review attends to a number of key factors that may influence effectiveness of these interventions. Specifically, our review suggests a benefit to involving a provider in the delivery process, as compared with technology-based alcohol interventions that only engage the patient. This aligns with much research on technology-based interventions for use of tobacco and other substances [75,76]. Extensive research has shown that the specification and use of implementation strategies improve outcomes such as the adoption, reach, and sustainability of interventions [77-80]. However, to the extent that studies reported the use of implementation strategies when they were used, our review is among the first to find that employing an implementation strategy may actually enhance the effectiveness of a behavioral health intervention.

Our systematic review also highlights a pattern of growth in the number of studies evaluating technology-based alcohol interventions in primary care, with nearly 3 times as many studies on technology-based alcohol interventions in primary care published during the years of 2010 to 2018, as compared with the years of 2000 to 2009. The increasing number of studies on these interventions reflects an important angle of the changing health care landscape. As innovative technology-based approaches to delivering alcohol interventions continue to rapidly develop, it is necessary to take stock of the existing efforts and identify areas for further growth and improvement. Results indicated robust potential of technology-based interventions to support alcohol-related behavior change, with the majority indicating reduced drinking or harm in all of the reported primary and secondary outcomes. Similarly, when compared directly with TAU, there appeared to be strong efficacy for technology-based alcohol interventions over and above nontechnology-based alcohol intervention, with 42% (13/31) of studies with direct comparisons with TAU reporting that at least half of the primary and secondary efficacy outcomes of the technology-based interventions were superior to TAU.

Our review also highlights factors that appear to influence intervention efficacy. For instance, we identified 4 broad categories of technology-based alcohol interventions, and results indicated that interventions with a provider-based component (particularly provider-facilitated and patient-facing plus provider-facilitated interventions) were more efficacious than those that were patient-facing only. These findings suggest that provider involvement in the delivery of technology-based alcohol interventions may boost efficacy; however, further research is needed in this area.

Another key finding was that studies describing use of an implementation strategy reported more effective technology-based alcohol interventions. Although this effect has been documented in limited previous research [81], this remains a relatively novel finding that contributes to the accumulating evidence of the value-added benefit of employing implementation strategies to facilitate EBIs. Nevertheless, this finding is consistent with theoretical advances in the implementation science field that reject the assumption of *voltage drop* as an intervention moves from efficacy trials to real-world implementation studies [82]. Instead, it is reasonable to expect that with active strategies to adapt and tailor interventions to contexts and patients, the *voltage* of a technology-based intervention may even be enhanced. We encourage further empirical study of this potential effect in future research.

Yet, the determinants of efficacy remain largely unexplained, even after accounting for the presence of a provider-based component and a specified implementation strategy. Intermediate outcomes, or implementation outcomes (eg, acceptability, feasibility, and sustainability) [83,84], which are often not reported on in clinical research may help to further explain intervention efficacy. For instance, the degree to which patients and providers find particular technology-based alcohol interventions to be acceptable, feasible, and sustainable to use may influence the effectiveness of those interventions.

Future research should also strive to report more frequently on implementation strategies that occur during intervention studies [65]. Although outside the scope of this study, future reviews may benefit from examining whether or not the effectiveness of technology-based interventions can be predicted by the type of implementation strategy used. For instance, it is conceivable that variation in training and ongoing technical assistance, mandates from leadership, or efforts to engage patients and increase consumer demand for technology-based alcohol interventions could lead to greater effectiveness of these interventions. Relatedly, implementation studies should continue to examine the systematic use of strategies to support or improve the delivery of technology-based alcohol interventions.

Finally, this review highlights a potential lack of current integration between technology-based alcohol interventions and existing EHR systems; this technological integration has been posited to be a critical limiting factor in realizing the public health impact promised by technology-based behavioral health interventions [85]. Greater efforts to integrate technology-based alcohol interventions with existing EHR systems in primary care will be necessary to ensure the scale up and sustainability of technology-based alcohol interventions.

### Limitations

We acknowledge that our systematic review may be limited to some degree by publication bias. It is important to recognize that there is a general bias, in both authors and publishers, toward prioritizing the publication of positive findings (ie, evidence in support of tested interventions) over null or negative findings. This can lead to misleadingly favorable conclusions about the efficacy of interventions. Although unable to fully address this limitation, we included dissertations in our systematic review to help mitigate this concern.

It is also worth noting that, given the nascent stage of the implementation science field, authors are often inconsistent in their reporting of implementation strategies used to deliver their interventions [65]. Some authors may not have reported or specified implementation strategies that were actually used, increasing the risk of reporting bias. Additionally, different authors may have specified the same implementation strategy in different ways. It is possible that these inconsistencies influenced our findings regarding the relationship between implementation strategies and efficacy.

As is generally the case in systematic reviews, our study is subject to the limitations of potential errors or biases in searching, including, and coding articles. For instance, it is possible that our search strategy failed to identify all relevant studies (eg, non-English language articles), that we were too conservative in our inclusion of relevant studies, or that we erroneously coded content in some articles. To offset these concerns, we (1) solicited the expertise of 2 university librarians to conduct extensive database and search string testing to ensure that our search strategy was appropriately comprehensive and (2) used 2 study investigators during the inclusion and coding processes to limit reviewer fatigue and ensure interrater agreement. Finally, we limited our review to primary care and general practice settings, and although previous reviews have addressed technology-based behavioral health interventions in the emergency department and social work settings [15,16,86], future research focused on other settings, including specialty care or dental health, may further contribute to this area of investigation.

### Implications

This systematic review contributes substantially to the conceptualization of technology-based alcohol interventions, an understanding of the range of implementation contexts and formats in which these interventions are delivered, and an initial assessment of the efficacy and effect modifiers for technology-based alcohol interventions. The use of technology-based tools in primary care settings represents a promising approach to enhance the efficiency, service delivery flexibility, and effectiveness of interventions for alcohol-related problems. Our systematic review identifies that the past 2 decades have borne witness to the delivery of technology-based alcohol interventions through a variety of different technological platforms (eg, computer, Web, or mobile), settings (eg, clinic, home, or both), and targets (eg, patients, providers, or both). These findings provide initial support for the efficacy of technology-based alcohol interventions, particularly when deployed through a specific implementation strategy and involving a provider in the delivery process, and we encourage future research to further establish the efficacy, moderators of efficacy, and implementation strategies for delivering these types of interventions in primary care settings and beyond.

## Appendix

Multimedia Appendix 1 Search string of 77 Boolean terms searched by Title, Abstract, and Subject.

Multimedia Appendix 2 Studies of technology-based alcohol interventions in primary care (n=42).

Multimedia Appendix 3 Summary of results on alcohol-related outcomes.

## Abbreviations

ANOVA: analysis of variance
EBI: evidence-based intervention
EHR: electronic health record
TAU: treatment as usual
