# Supplementary material for: Technology-Based Alcohol Interventions in Primary Care: Systematic Review
Source: J Med Internet Res. 2019 Apr 8;21(4):e10859. doi: 10.2196/10859 (PMC6475823; doi:10.2196/10859)
Supplement: Multimedia Appendix 1 [file jmir_v21i4e10859_app1.pdf]

Multimedia Appendix 1. Search string of 77 Boolean terms searched by Title, Abstract, and Subject.

Primary Care

"primary care" OR "primary health care" OR "primary healthcare" OR "family medicine" OR "family practice“ OR "general practice" OR "physicians, Family" OR "general practitioners"

Technology

technolog* OR wireless OR electronic* OR computer* OR mobile OR web OR website* OR internet* OR digital* OR virtual* OR online OR apps OR application* OR video* OR gaming OR smartphone* OR "smart phone*" OR ipad* OR laptop* OR tablet* OR "social network*" OR "social media" OR twitter OR youtube OR facebook OR texting OR "text messag*" OR "app" OR "iphone*" OR "apple watch" OR "google glass*” OR "wii" OR kiosk* OR "eHealth*" OR "e-health*" OR mHealth* OR telemedicine OR software* OR telehealth* OR multimedia OR "m-health*" OR "geograph* information system*" OR "remote consultation*" OR "gis“ OR "short messag* service" OR "voice recognition" OR "global positioning system*" OR "speech recognition" OR "interactive voice response“ OR "cell phone*" OR cellphone* OR telephone* OR "cellular phone*"

Alcohol

alcohol* OR drinking OR "driving while drunk" OR "driving drunk" OR "drunk* driving" OR "driving under the influence“ OR "driving while intoxicated" OR "DUI" OR "DWI" OR drunkenness OR "impaired driving" OR "drugged driving" OR "driving while impaired“ OR "driving impaired" OR "driving intoxicated" OR "intoxicated driving"
