# Supplementary material for: Technology-Based Alcohol Interventions in Primary Care: Systematic Review
Source: J Med Internet Res. 2019 Apr 8;21(4):e10859. doi: 10.2196/10859 (PMC6475823; doi:10.2196/10859)
Supplement: Multimedia Appendix 2 [file jmir_v21i4e10859_app2.pdf]

Multimedia Appendix 2. Studies of technology-based alcohol interventions in primary care (n=42).

**Study Identifier**	**Study Design/ Sample Size**	**Technology-based intervention (*underlying evidence-based practice*)**	**Target of Technology**	**Delivery Context**	**Implementation Strategies {*Expert Recommendations for Implementing Change (ERIC) Categorization*}**	**Primary/ Secondary Outcomes**	**Intervention Efficacy/ Main Results**	**Risk of Bias (0 to 10 scale)**
Acosta 2017	RCT<br><br>n=162 veteran adult patients	Self-directed, web-based interactive modules *(Cognitive Behavioral Theory)*	Patient-facing	In clinic and at home	None specified	% drinking days and % of heavy drinking days during in-intervention and 3-month post-intervention time periods.	Significantly greater declines in % heavy drinking days in intervention group vs. treatment as usual. This difference remained at post-intervention assessment.	1
Bendtsen 2011	Quasi-experimental; One group pre-post<br><br>n=334 adult patients	Stand-alone touchscreen kiosk electronic screening and brief intervention, with printed patient feedback report (*Motivational Interviewing*)	Patient-facing	In clinic	Self-referred vs staff-referred administration of electronic screening and brief intervention {*Prepare patients / consumers to be active participants*}	Weekly consumption, grams/week; heavy episodic drinking occasions per month / % changed from risk to no risk	Significant reduction in heavy episodic drinking, significant reduction in weekly alcohol consumption for staff-referred group (not self-referred group)	7
Bischof 2008	RCT<br><br>n=408 adult patients	Computer-based feedback intervention (*Transtheoretical Model*) + telephone-based counseling sessions (*Motivational Interviewing*)	Patient-facing + Provider-facilitated	In clinic and at home	Counselors training with manual, weekly supervision sessions {*Conduct ongoing training*}	Change in grams/day consumption; % binge / % help-seeking; duration of counseling sessions	Significant decrease in alcohol per day, small-to-medium effect in those with abuse and/or at-risk; savings of 13.5-22.23 euros per patient	0
Brown 2007	RCT<br><br>n=897 adult patients	Telephone counseling (*Motivational Interviewing*)	Provider-facilitated	At home	Counselors training with manual, biweekly supervision {*Conduct ongoing training*}	Total consumption and days of at-risk consumption	Significant reductions in total consumption and risky drinking days for men; non-significant reductions in women	0
Butler 2003	Quasi-experimental; Staggered rollout, sites serving as own controls	Kiosk equipped with a touch screen monitor, computer, and printer; generates tailored feedback report	Patient-facing	In clinic	None specified	Quantity and frequency of alcohol use (first 3 questions of AUDIT) / alcohol discussions with provider	Null findings for alcohol use reduction and alcohol discussions; significant difference over 6 month follow-up but not significant intervention effect	2

	n=128 adult patients							
Cucciare 2013	RCT<br><br>n=167 veterans	Web-based brief alcohol intervention	Patient-facing	In clinic	None specified	% of heavy drinking days; Mean drinks per drinking day; total drinking days; Severity of alcohol-related Problems	Both intervention and control groups showed significant reduction; no group difference	3
Dawson-Rose 2017	RCT<br><br>n=208 HIV+ adult patients	Self-administered computerized SBIRT procedure embedded in clinic personal health record (*Motivational Interviewing)*	Patient-facing + provider-facilitated	In clinic	None specified	Frequency and severity of alcohol use as measured by Specific Substance Involvement Score (SSIS) using ASSIST.	No between-group differences in SSIS over time.	1
Dickinson 2013	RCT<br><br>n=169 patients	Interactive web-based intervention with action planning component	Patient-facing	At home	None specified	Heavy drinking days in past 30 days	Not enough positive screens to detect change over time	1
Dimeff 2000	RCT<br><br>n=41 students	Interactive computer-based program with printed patient feedback report (*Motivational Interviewing*)	Patient-facing + provider-facilitated	In clinic	Use of acronyms (SOW and REAP) to help train providers to use intervention {*Develop educational materials*}	Binge episodes in past week; alcohol problems experienced in past 30 days	No between group differences found; Moderate to large effect size in favor of intervention	5
Duru 2015	RCT; cluster-randomized<br><br>n=31 physicians<br>n=1,186 patients aged 60+	Computer-generated individualized risk reports provided by physicians; telephone-based counseling from health educator	Provider-facilitated	In clinic and at home	None specified	At-risk drinking in the last 6 months as measured by CARET survey items.	Significantly lower likelihood of at-risk drinking among intervention group if they followed telephone-counseling suggestions of making a drinking agreement and/or keeping a drinking diary at both 6 and 12-months.	0
Fink 2005	Site RCT<br><br>n=665 older adults	Computer-based screening and education program w/ patient feedback report	Patient-facing + provider-facilitated	In clinic and at home	None specified	Drinking classification (hazardous/harmful) / Drinks per week	Significant reductions in hazardous and harmful drinking; significant decrease in quantity and frequency in the provider + patient group	2

Gryczynski 2015	RCT<br><br>n=360 adults	Tablet computer w/ avatar	Patient-facing	In clinic	None specified	Alcohol risk score on the ASSIST	Significant reduction of alcohol risk in computer group (compared to in-person) at 3 months; converged at 6 and 12 months.	2
Harris 2012	Quasi-experimental; One group pre-post<br><br>n=2096 (New England) + 589 (Prague) adolescents	Computer screen, computer feedback and brief intervention; providers receive results and talking points	Patient-facing + provider-facilitated	In clinic	Gave providers computerized screening results {*Relay clinical data to providers*}	% of any past 90 day alcohol use at 3 months and % of any past 12 month alcohol use at 12 months / satisfaction with visit and perception of counseling behaviors	Significant reduction (compared to treatment as usual) in alcohol use in New England at 3 and 12 months; more positive ratings of visit and perception of counseling behaviors as well	6
Hasin 2013	RCT<br><br>n=258 adults	Interactive voice response; automated telephone system w/ digital alarm watch and personalized feedback (*Motivational Interviewing*)	Patient-facing + provider-facilitated	In clinic and at home	None specified	Drinks per drinking day in last 30 days / Interaction of treatment X alcohol dependence on drinking days; % days abstinent	Significant reductions in number of drinks in MI+Healthcall group; stronger in alcohol dependent patients	1
Helstrom 2014	RCT<br><br>n=139 adults	Telephone-based brief intervention	Provider-facilitated	At home	None specified	Drinking days in the past month, binges, drinks per drinking day / problems due to alcohol, motivation to change	Both treatment and comparison groups reduced drinking; 40% no longer heavy drinking in treatment group	1
Helzer 2008	RCT<br><br>n=338 adults	Interactive voice response + patient feedback report to enhance brief intervention	Patient-facing	At home	None specified	Drinks per week / drinking days per week, drinks per drinking day, % currently dependent	Mixed results; no interactive voice response (IVR) group performed better, but measurement confound; IVR + feedback performed better than IVR w/o feedback	4
Holtrop 2008	Quasi-experimental; One group pre-post<br><br>n=15 practices; n=797 referrals	Telephone contact by referral liaison	Provider-facilitated	At home	Two levels of support: Referral-only with information / encouragement to refer, and consultant-enhancement with multiple visits from CHERL	Alcoholic drinks/occasion; Number of times alcohol drinks >5/occasion in the past month	Significant decrease in drinks/occasion	5

					{*Provide ongoing consultation*}			
Hunter 2017	Randomized non-inferiority trial<br><br>n=763 patients	Facilitated access to web-based intervention for hazardous drinking	Patient-facing + Provider-facilitated	In clinic and at home	None specified	Number of cases of hazardous or harmful drinking prevented	No significant between-group differences in number of hazardous or harmful drinking,	2
Kalapatapu 2014	Secondary analysis of data from RCT<br><br>n=103 patients	Telephone-administered treatment (*Cognitive-Behavioral Therapy*)	Provider-facilitated	At home	None specified	Change in AUDIT score	Both groups decreased in alcohol use at end of treatment; non-significant difference between groups	3
Knight 2018	Quasi-experimental; One group pre-post<br><br>n=2096 (New England) + 589 (Prague) adolescents	Computer screen, computer feedback and brief intervention; providers receive results and talking points	Patient-facing + provider-facilitated	In clinic	Gave providers computerized screening results {*Relay clinical data to providers*}	% of any past-90-day heavy episodic drinking at 3- and 12-months	Significant reduction (compared to treatment as usual) in heavy episodic drinking at 3 months; no effect at 12 months	6
Kypri 2008	RCT<br><br>n=576 students	Web-based electronic screening and brief intervention	Patient-facing	In clinic	None specified	Drinking days in past 2 weeks, drinks per drinking occasion in past 4 weeks, total drinks in past 2 weeks, episodes of heavy drinking in past 2 weeks, alcohol problems, consequences related to academic performance, AUDIT score at 12 months	Significant reductions in frequency, total consumption, AUDIT scores, heavy episodic drinking	1
Kypri 2004	RCT<br><br>n=104 students	Web-based electronic screening and brief intervention	Patient-facing	In clinic	None specified	Drinking days in past 2 weeks, drinks per drinking occasion in past 4 weeks, total drinks in past 2 weeks, episodes of heavy drinking in past 2 weeks, alcohol problems, consequences related to academic performance	Significant reductions in total consumption, lower heavy episodic drinking frequency, and consequences at 6 weeks; consequences lower at 6 months	2

Lin 2010	Secondary analysis of data from RCT<br><br>n=310 patients in intervention arm	Telephone-based intervention by health educators (*Motivational Interviewing*)	Provider-facilitated	At home	None specified	Alcohol-related risks	Conducting all three health educator calls increased odds of achieving not at-risk status compared to completing no calls at 3 months	3
McCausland 2011	RCT<br><br>n=43 adults	Computer-delivered patient feedback report	Patient-facing	In clinic	None specified	Drinks per week; Binge drinking episodes; harmful effects due to alcohol consumption in past 3 months	Significant changes in drinking norms and normative discrepancy, but not for drinking outcomes; significant reductions in weekly binge episodes and motivation to change alcohol consumption for men only.	0
McMenamin 2011	Observational<br><br>n=35 practices	Patient dashboard clinical management tool	Provider-directed	In clinic	Staff training and support with software via practice facilitators {*Facilitation*}; Link existing clinical management tools to dashboard as reminder system {*Remind clinicians*}	Screening rates for alcohol use	Recorded alcohol use screening rates increased from 15% to 47% over 15 month period	6
Moore 2011	RCT<br><br>n=631 patients	Telephone-counseling by health educator (*Motivational Interviewing*)	Provider-facilitated	At home	None specified	% at-risk drinkers / drinks in past 7 days, % heavy drinking in past 7 days	Significant reduction at 3 months in at-risk status, drinks, heavy drinking, risk scores; drinks at 12 months	3
Olson 2009	Quasi-experimental; One group pre-post<br><br>n=163 youths	Personal digital assistant (PDA) screening tool to enhance health behavior counseling	Patient-facing + provider-facilitated	In clinic	None specified	Patient-provider discussions of alcohol use and helpfulness of discussion / appraisal of visits	Pre-to-post discussion of alcohol use increased from 38% to 53.9%; no difference in perceived helpfulness of discussion	4
Oslin 2003	RCT<br><br>n=97 veterans	Telephone contact by behavioral health specialist to assist in care management	Provider-facilitated	At home	Behavioral health specialists met weekly with psychiatrist {*Organize clinician implementation team meetings*}	At-risk drinking response (average drinks per week and average binges in 3 months)	Significant improvement in telephone group vs. usual care in overall treatment response; alcohol-specific response to treatment was higher but non-significant	0
Pereira 2015	Quasi-experimental; One group pre-post	E-learning course: web conferences, audio/video recordings	Provider-directed	At home	Varied array of interactive e-learning tools for healthcare	Knowledge critical to management of alcohol problems, misconceptions	Significant improvement on critical knowledge, but not misconceptions; course	5

	n=33 completers				professionals {*Make training dynamic*}	in treating patients with alcohol problems	satisfaction was high	
Possemato 2013	Quasi-experimental; One group pre-post<br><br>n=1820 patients	Telehealth phone call and patient summary put in electronic medical record for provider review	Provider-facilitated	At home	None specified	Frequency of behavioral health care visits; AUDIT-C scores	Used significantly more health services and had lower alcohol screening scores after intervention	4
Quanbeck 2018	Quasi-experimental; Non-concurrent multiple-baseline design<br><br>n=268 patients	mHealth system to facilitate addiction treatment in primary care (*Cognitive-Behavioral Therapy*)	Patient-facing + provider-facilitated	In clinic and at home	Detailed, tailored implementation plans that included: assessments of readiness for implementation, training; rapid-cycle feedback; organizational coach {*Develop a formal implementation blueprint / Assess for readiness / Conduct ongoing training / Conduct cyclical small tests of change / Facilitation* }	Reach (patients enrolled); Effectiveness (risky drinking days in the past 30 days); Adoption (use of mHealth system by patients and providers); Implementation (stages completed, costs); and Maintenance (sustained effect at 6 months, sustained use at 12 months) of the mHealth system	Significant improvements (44% reduction) in risky drinking days; 53-60% of the patients accessed the mHealth system during the last week of the 12-month implementation period	4
Rose 2017	RCT<br><br>n=1855 patients	Interactive voice response + brief intervention	Patient-facing	At home	None specified	Drinks per week, drinking days per week, drinks per drinking day, heavy episodic drinking	No differences between IVR-BI and No IVR-BI groups at 3 or 6 months. Within-subject reductions in drinking over time were observed in individuals with AUD diagnosis.	2
Ruf 2010	Cluster RCT<br><br>n=112 practices	E-learning, online quality improvement program	Provider-directed	At home	Training for physicians and nurses followed by phone contact to solve ongoing problems {*Provide ongoing consultation*}	Change in the severity of the alcohol problem after 3 months / Acceptance and use of the system; % of correct diagnoses, correct treatment and referral rate	No group differences in use of the system; 41.4-72.7% of patients had reduced severity of alcohol problem (depending on dissemination strategy)	2

Schwartz 2014	RCT<br><br>n=360 patients	Computerized brief intervention	Patient-facing	In clinic	None specified	ASSIST scores (global and alcohol-specific)	Non-significant improvements in computerized brief intervention (compared to in-person intervention) in global ASSIST and alcohol-specific scores	1
Stoner 2014	RCT<br><br>n=92 providers	Web-based multimedia training on screening, brief intervention, and referral to treatment	Provider-directed	At home	Multimedia training for physicians, physician assistants, and nurse practitioners {*Make training dynamic*}	Self-efficacy, clinical practice behaviors, clinical practice behavioral intentions, knowledge, training completion and satisfaction	Similar to comparison group (online reading materials), but more likely to be completed and rated more favorably.	0
Tanner 2012	Quasi-experimental; One group pre-post n=70 students, n=65 providers	Web-based interactive skills training program	Provider-directed	At home	Interactive training for health professional students and primary care providers {*Make training dynamic*}	Knowledge, self-efficacy, clinical comprehension, satisfaction	Preliminary findings suggest that all outcomes improved after training intervention; *p*-values not reported.	6
Vinson 2000	RCT<br><br>n=80 patients	Computer-generated written behavioral contract	Patient-facing + Provider-facilitated	In clinic	None specified	AUDIT and ASI scores / hazardous drinking days in previous 12 weeks; perceived acceptability and feasibility	Alcohol risk decreased non-significantly in intervention group (compared to control) at 12 months; acceptable and feasible	1
Walton 2013	RCT<br><br>n=328 adolescents	Tablet-based interactive animated program/ brief intervention	Patient-facing	In clinic	None specified	Frequency of alcohol use	No significant group differences for alcohol use at 3 or 6 months	1
Walton 2014	RCT<br><br>n=714 adolescents	Computer-based interactive animated program/ brief intervention	Patient-facing	In clinic	None specified	Alcohol use severity	Non-significant decrease in alcohol use severity at 6 months in computerized brief intervention group	1
Williams 2010	Secondary analysis of group RCT<br><br>n=1358 patients	Electronic clinical reminder to facilitate brief alcohol interventions	Provider-directed	In clinic	Electronic clinical reminder {*Remind clinicians*}	% of documented brief intervention use; severity of unhealthy alcohol use	39% of patients w/ clinical-reminder use had documented brief intervention; access to reminder not significantly associated with resolution of unhealthy drinking	4
Wongparakan 2011	RCT<br><br>n=54 participants	Telephone-based intervention (*Motivational*	Provider-facilitated	At home	None specified	Change in amount of alcohol use; drinking days	Significantly lower amount and frequency of drinking in intervention group	1

		Interviewing)						
Zanjani 2010	RCT<br><br>n=113 patients	Telephone-based referral-care management intervention	Provider-facilitated	At home	None specified	Total drinks in last week; binge rate in last 3 months	Significantly improved binge outcomes in both groups over 6 months; no significant difference between intervention and control groups.	1

RCT=Randomized controlled trial

Risk of Bias=Score assigned on 0 to 10 scale upon judging high (2), uncertain (1), or low (0) risk among five domains of bias: selection, performance, detection, attrition, and reporting.
