# Supplementary material for: Technology-Based Alcohol Interventions in Primary Care: Systematic Review
Source: J Med Internet Res. 2019 Apr 8;21(4):e10859. doi: 10.2196/10859 (PMC6475823; doi:10.2196/10859)
Supplement: Multimedia Appendix 3 [file jmir_v21i4e10859_app3.pdf]

Multimedia Appendix 3. Summary of results on alcohol-related outcomes.

Tech type	Alcohol-related outcome	Study	Significance level (*P* value)	
			Reduce drinking or harms?	Superior to TAU?
**Patient-facing[a]**				
	**Quantity of alcohol use**			
		Bendtsen 2011 [23]	Yes (for staff-referred patients)*<br>NS (for self-referred patients)	N/A
		Cucciare 2013 [27]	NS at 3 months[b]<br>Yes at 6 months*	NS
		Helzer 2008 [35]	Yes*	No (*inferior* to TAU)*
		Kypri 2008 [12] (total quantity)	—[c]	Yes at 6 months*<br>Yes at 12 months*
		Kypri 2008 [12] (typical quantity)	—	NS at 6 months<br>NS at 12 months
		Kypri 2004 [38] (total quantity)	—	Yes at 6 weeks*<br>NS at 6 months
		Kypri 2004 [38] (typical quantity)	—	NS at 6 weeks<br>NS at 6 months
		McCausland 2011 [40]	—	NS
		Rose 2017 [63]	Yes**	NS
	**Frequency of alcohol use**			
		Acosta 2017 [57]	Yes at 3 months*	Yes at 6 months*
		Cucciare 2013 [27]	Yes at 3 months*<br>Yes at 6 months*	NS

		Kypri 2008 [12]	—	Yes at 6 months** NS at 12 months[b]
		Kypri 2004 [38]	—	NS at 6 weeks [b] NS at 6 months
		Rose 2017 [63]	Yes**	NS
		Walton 2013 [52]	Yes**	NS
	**Severity of alcohol use or risk scores**			
		Butler 2003 [26]	Yes***	NS
		Cucciare 2013 [27]	Yes at 3 months* Yes at 6 months*	NS
		Gryczynski 2015 [31]	Yes***	Yes*
		Kypri 2008 [12]	—	Yes at 12 months***
		Schwartz 2014 [48]	—	NS
		Walton 2014 [53]	—	NS
	**Binge or heavy episodic drinking**			
		Bendtsen 2011 [23]	Yes***	N/A
		Cucciare 2013 [27]	Yes at 3 months* Yes at 6 months*	NS
		Dickinson 2013 [28]	—[d]	N/A
		Kypri 2008 [12]	—	NS at 6 months; NS at 12 months
		Kypri 2004 [38]	—	Yes at 6 weeks* NS at 6 months
		McCausland 2011 [40]	—	NS [b]
		Rose 2017 [63]	Yes**	NS
	**Drinking consequences**			

		Kypri 2008 [12] (personal, social, sexual, or legal)	—	NS at 6 months
				NS at 12 months [b]
		Kypri 2008 [12] (academic)	—	Yes at 6 months**
				Yes at 12 months*
		Kypri 2004 [38] (personal, social, sexual, or legal)	—	Yes at 6 weeks* Yes at 6 months*
		Kypri 2004 [38] (academic)	—	NS at 6 weeks [b] NS at 6 months [b]
		McCausland 2011 [40]	—	NS
**Provider-facilitated[e]**				
	**Quantity of alcohol use**			
		Brown 2007 [25]	Yes (men)***	Yes (men)***
			Yes (women)***	NS (women)
		Helstrom 2014 [34]	Yes***	NS
		Holtrop 2008 [36]	Yes***	N/A
		Moore 2011 [42]	Yes at 3 months* Yes at 12 months*	Yes at 3 months*** Yes at 12 months*
		Wongpakaran 2011 [55]	Yes**	Yes**
		Zanjani 2010 [56]	NS	NS
	**Frequency of alcohol use**			
		Brown 2007 [25]	Yes (men)*** Yes (women)***	Yes (men)*** NS (women)

		Helstrom 2014 [34]	Yes***	NS
		Wongparakan 2011 [55]	Yes**	Yes**
	**Severity of alcohol use or risk scores**			
		Kalapatapu 2014 [37]	Yes***	NS
		Moore 2011 [42]	Yes at 3 months*<br>Yes at 12 months*	Yes at 3 months**<br>NS at 12 months
		Possemato 2013 [46]	Yes*	N/A
	**Binge or heavy episodic drinking**			
		Duru 2015 [59]	—	Yes at 6 months*<br>Yes at 12 months*
		Holtrop 2008 [36]	NS	N/A
		Moore 2011 [42]	Yes at 3 months*<br>Yes at 12 months*	Yes at 3 months*<br>NS at 12 months
		Zanjani 2010 [56]	Yes*	NS
	**Status, at-risk alcohol use**			
		Lin 2010 [39]	Yes at 3 months*<br>NS at 12 months	Yes at 3 months**<br>NS at 12 months
		Moore 2011 [42]	Yes at 3 months*<br>Yes at 12 months*	Yes at 3 months**<br>NS at 12 months
		Oslin 2003 [44]	—	Yes*
**Patient-facing plus provider-facilitated[f]**				
	**Quantity of alcohol use**			
		Bischof 2008 [24]	Yes*	Yes*
		Fink 2005 [30]	Yes*	Yes*

		Hasin 2013 [33]	Yes**	NS
	Frequency of alcohol use	Quanbeck 2018 [62]	Yes*	Yes*
	**Severity of alcohol use or risk scores**			
		Dawson-Rose 2017 [58]	Yes*	NS
		Vinson 2000 [51]	—	NS
	**Binge or heavy episodic drinking**			
		Bischof 2008 [24]	NS	NS
		Dimeff 2000 [29]	—	—
		Knight 2018 [61]	Yes at 3 months*<br>NS at 12 months	Yes at 3 months*<br>NS at 12 months
	**Status, at-risk alcohol use**			
		Fink 2005 [30]	Yes*	Yes*
		Hunter 2017 [60]	—	NS
	Any Use	Harris 2012 [32]	Yes at 3 months*<br>Yes at 12 months*	Yes at 3 months*<br>Yes at 12 months*
**Provider-directed[g]**				
	**Severity of alcohol use or risk scores**	Ruf 2010 [47]	—	N/A
		Williams 2010 [54]	—	NS

[a]Patient-facing: Intervention delivered directly to patient via technology with very limited or no provider involvement.

[b]NS but trend of *P*<.10.

[c]Empty cells (—) reflect missing data or not applicable to referenced article.

[d]Too few drinkers at baseline to detect change.

[e]Provider-facilitated: Intervention delivered by provider to patient via technology-based medium.

[f]Patient-facing plus provider-facilitated: Packaged intervention that has at least one patient-facing component and at least one provider-facilitated component.

[g]Provider-directed: Intervention is delivered to provider to improve or support patient care delivery.

**P*<.05

***P*<.01

****P*<.001

12. Kypri K, Langley JD, Saunders JB, Cashell-Smith ML, Herbison P. Randomized controlled trial of web-based alcohol screening and brief intervention in primary care. Arch Intern Med. 2008 Mar 10;168(5):530–6. PMID:18332300

23. Bendtsen P, Ekman DS, Johansson A, Carlfjord S, Andersson A, Leijon M, et al. Referral to an Electronic Screening and Brief Alcohol Intervention in Primary Health Care in Sweden: Impact of Staff Referral to the Computer. Int J Telemed Appl. 2011 Jan;2011:2:1–2:10. PMID:21603024

24. Bischof G, Grothues JM, Reinhardt S, Meyer C, John U, Rumpf H-J. Evaluation of a telephone-based stepped care intervention for alcohol-related disorders: A randomized controlled trial. Drug Alcohol Depend. 2008 Mar 1;93(3):244–51. PMID:18054443

25. Brown RL, Saunders LA, Bobula JA, Mundt MP, Koch PE. Randomized-Controlled Trial of a Telephone and Mail Intervention for Alcohol Use Disorders: Three-Month Drinking Outcomes. Alcohol Clin Exp Res. 2007 Aug 1;31(8):1372–9. PMID:17550366

26. Butler SF, Chiauzzi E, Bromberg JI, Budman SH, Buono DP. Computer-Assisted Screening and Intervention for Alcohol Problems in Primary Care. J Technol Hum Serv. 2003 Jun 1;21(3):1–19. DOI:10.1300/J017v21n03_01

27. Cucciare MA, Weingardt KR, Ghaus S, Boden MT, Frayne SM. A randomized controlled trial of a web-delivered brief alcohol intervention in Veterans Affairs primary care. J Stud Alcohol Drugs. 2013;74:428-36. PMID:23490572

28. Dickinson WP, Glasgow RE, Fisher L, Dickinson LM, Christensen SM, Estabrooks PA, et al. Use of a Website to Accomplish Health Behavior Change: If You Build It, Will They Come? And Will It Work If They Do? J Am Board Fam Med. 2013 Mar 1;26(2):168–76. PMID:23471930

29. Dimeff LA, McNeely M. Computer-enhanced primary care practitioner advice for high-risk college drinkers in a student primary health-care setting. Cogn Behav Pract. 2000;7(1):82–100. DOI:0.1016/S1077-7229(00)80010-3

30. Fink A, Elliott MN, Tsai M, Beck JC. An evaluation of an intervention to assist primary care physicians in screening and educating older patients who use alcohol. J Am Geriatr Soc. 2005;53:1937-43. PMID:16274375

31. Gryczynski J, Mitchell SG, Gonzales A, Moseley A, Peterson TR, Ondersma SJ, et al. A randomized trial of computerized vs. in-person brief intervention for illicit drug use in primary care: Outcomes through 12 months. J Subst Abuse Treat. 2015 Mar;50:3–10. PMID:25282578

32. Harris SK, Csémy L, Sherritt L, Starostova O, Hook SV, Johnson J, et al. Computer-Facilitated Substance Use Screening and Brief Advice for Teens in Primary Care: An International Trial. Pediatr. 2012 Jun 1;129(6):1072–82. PMID:22566420

33. Hasin DS, Aharonovich E, O'Leary A, Greenstein E, Pavlicova M, Arunajadai S, et al. Reducing heavy drinking in HIV primary care: a randomized trial of brief intervention, with and without technological enhancement. Addict. 2013 Jul 1;108(7):1230–40. PMID:23432593

34. Helstrom AW, Ingram E, Wang W, Small D, Klaus J, Oslin D. Treating Heavy Drinking in Primary Care Practices: Evaluation of a Telephone-based Intervention Program. Addict Disord Their Treat. 2014 Sep;13(3):101–9. DOI:10.1097/ADT.0b013e31827e206c

35. Helzer JE, Rose GL, Badger GJ, Searles JS, Thomas CS, Lindberg SA, et al. Using Interactive Voice Response to Enhance Brief Alcohol Intervention in Primary Care Settings. J Stud Alcohol Drugs. 2008;69:251-8. PMID:18299766

36. Holtrop JS, Dosh SA, Torres T, Thum YM. The Community Health Educator Referral Liaison (CHERL): A Primary Care Practice Role for Promoting Healthy Behaviors. Am J Prev Med. 2008 Nov;35(5, Supplement):S365–72. PMID:18929983

37. Kalapatapu RK, Ho J, Cai X, Vinogradov S, Batki SL, Mohr DC. Cognitive-Behavioral Therapy in Depressed Primary Care Patients with Co-Occurring Problematic Alcohol Use: Effect of Telephone-Administered vs. Face-to-Face Treatment—A Secondary Analysis. J Psychoactive Drugs. 2014 Mar 15;46(2):85–92. PMID:25052784

38. Kypri K, Saunders JB, Williams SM, McGee RO, Langley JD, Cashell-Smith ML, et al. Web-based screening and brief intervention for hazardous drinking: a double-blind randomized controlled trial. Addict. 2004 Nov 1;99(11):1410–7. PMID:15500594

39. Lin JC, Karno MP, Tang L, Barry KL, Blow FC, Davis JW, et al. Do Health Educator Telephone Calls Reduce At-risk Drinking Among Older Adults in Primary Care? J Gen Intern Med. 2010 Jan 26;25(4):334–9. PMID:20101471

40. McCausland C. A randomized clinical trial of computer delivered personalized feedback for hazardous drinkers in primary care. 2011. Unpublished Dissertation. The University of Memphis.

42. Moore AA, Blow FC, Hoffing M, Welgreen S, Davis JW, Lin JC, et al. Primary care-based intervention to reduce at-risk drinking in older adults: a randomized controlled trial. Addict. 2011 Jan 1;106(1):111–20. PMID:21143686

44. Oslin DW, Sayers S, Ross J, Kane V, Ten Have T, Conigliaro J, et al. Disease management for depression and at-risk drinking via telephone in an older population of veterans. Psychosom Med. 2003 Dec;65(6):931–7. PMID:14645769

46. Possemato K, Bishop TM, Willis MA, Lantinga LJ. Healthcare Utilization and Symptom Variation Among Veterans Using Behavioral Telehealth Center Services. J Behav Health Serv Res. 2013 Apr 25;40(4):416–26. PMID:23616251

47. Ruf D, Berner M, Kriston L, Lohmann M, Mundle G, Lorenz G, et al. Cluster-randomized Controlled Trial of Dissemination Strategies of an Online Quality Improvement Programme for Alcohol-related Disorders. Alcohol Alcohol. 2010 Jan 1;45(1):70–8. PMID:19889887

48. Schwartz RP, Gryczynski J, Mitchell SG, Gonzales A, Moseley A, Peterson TR, et al. Computerized versus in-person brief intervention for drug misuse: a randomized clinical trial. Addict. 2014 Jul 1;109(7):1091–8. PMID:24520906

51. Vinson DC, Devera-Sales A. Computer-Generated Written Behavioral Contracts with Problem Drinkers in Primary Medical Care. Subst Abus. 21(4):215–22. PMID:12466660

52. Walton MA, Bohnert K, Resko S, Barry KL, Chermack ST, Zucker RA, et al. Computer and therapist based brief interventions among cannabis-using adolescents presenting to primary care: One year outcomes. Drug Alcohol Depend. 2013 Oct 1;132(3):646–53. PMID:23711998

53. Walton MA, Resko S, Barry KL, Chermack ST, Zucker RA, Zimmerman MA, et al. A randomized controlled trial testing the efficacy of a brief cannabis universal prevention program among adolescents in primary care. Addict. 2014 May 1;109(5):786–97. PMID:24372937

54. Williams EC, Achtmeyer CE, Kivlahan DR, et al. Evaluation of an Electronic Clinical Reminder to Facilitate Brief Alcohol-Counseling Interventions in Primary Care. J Stud Alcohol Drugs. 2010;71:720-5. PMID:20731977

55. Wongpakaran T, Petcharaj K, Wongpakaran N, et al. The effect of telephone-based intervention (TBI) in alcohol abusers: a pilot study. J Med Assoc Thai. 2011;94:849-56. PMID:21774293

56. Zanjani F, Bush H, Oslin D. Telephone-Based Psychiatric Referral-Care Management Intervention Health Outcomes. Telemed E Health. 2010 Jun 1;16(5):543–50. PMID:20575721

57. Acosta MC, Possemato K, Maisto SA, Marsch LA, Barrie K, Lantinga L, et al. Web-delivered CBT reduces heavy drinking in OEF-OIF veterans in primary care with symptomatic substance use and PTSD. Behav Ther. 2017;48(2), 262-276. PMID:28270335

58. Dawson-Rose C, Draughon JE, Cuca Y, Zepf R, Huang E, Cooper BA, et al. Changes in Specific Substance Involvement Scores among SBIRT recipients in an HIV primary care setting. Addict Sci Clin Pract. 2017;12(1), 34. PMID:29229000

59. Duru OK, Xu H, Moore AA, Mirkin M, Ang A, Tallen L, et al. Examining the impact of separate components of a multicomponent intervention designed to reduce at-risk drinking among older adults: The Project SHARE Study. Addict Sci Clin Pract. 2015;39(7), 1227-1235. PMID:26033430

60. Hunter R, Wallace P, Struzzo P, Della Vedova R, Scafuri F, Tersar C, et al. Randomised controlled non-inferiority trial of primary care-based facilitated access to an alcohol reduction website: cost-effectiveness analysis. BMJ Open. 2017;7(11), e014577. PMID:29102983

61. Knight JR, Kuzubova K, Csemy L, Sherritt L, Copelas S, Harris SK. Computer-facilitated screening and brief advice to reduce adolescents' heavy episodic drinking: a study in two countries. J Adolesc Health. 2018;62(1), 118-120. PMID:29054734

62. Quanbeck A, Gustafson DH, Marsch LA, Chih MY, Kornfield R, McTavish F, et al. Implementing a mobile health system to integrate the treatment of addiction into primary care: a hybrid implementation-effectiveness study. J Med Internet Res. 2018;20(1). PMID:29382624

63. Rose GL, Badger GJ, Skelly JM, MacLean CD, Ferraro TA, Helzer JE. A randomized controlled trial of brief intervention by interactive voice response. Alcohol Alcohol. 2017;52(3), 335-343. PMID:28069598
